# Supplementary material for: Quinoxaline-Based Anti-Schistosomal Compounds Have Potent Anti-Malarial Activity
Source: bioRxiv. 2024 Apr 24:2024.04.23.590861. Preprint. [Version 1] doi: 10.1101/2024.04.23.590861 (PMC11071471; doi:10.1101/2024.04.23.590861)
Supplement: Supplement 1 [file NIHPP2024.04.23.590861v1-supplement-1.pdf]

bioRxiv preprint doi: https://doi.org/10.1101/2024.04.23.590861; this version posted April 24, 2024. The copyright holder for this preprint (which was not certified by peer review) is the author/funder, who has granted bioRxiv a license to display the preprint in perpetuity. It is made available under aCC-BY 4.0 International license.

Compound 22

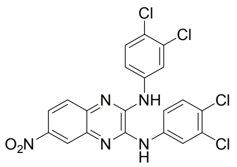

Compound 22c

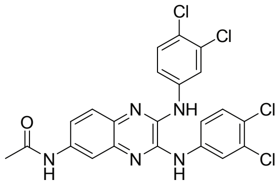

Compound 22f

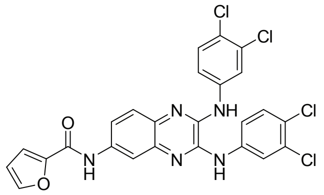

Compound 25

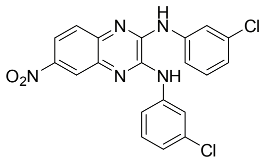

Compound 26

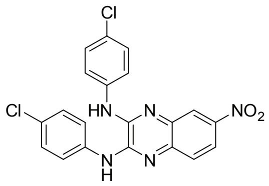

Compound 30

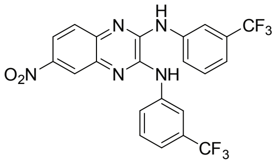

Compound 31

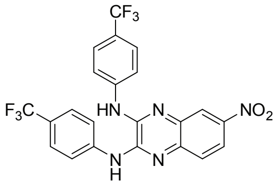

Compound 32

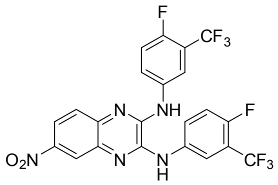

Compound 33

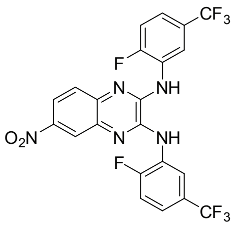

Compound 35

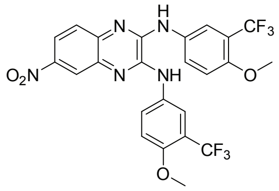

Compound 37

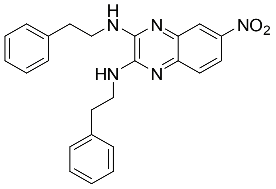

Supplementary Figure 1: Structures of compounds

bioRxiv preprint doi: https://doi.org/10.1101/2024.04.23.590861; this version posted April 24, 2024. The copyright holder for this preprint (which was not certified by peer review) is the author/funder, who has granted bioRxiv a license to display the preprint in perpetuity. It is made available under aCC-BY 4.0 International license.

A

Organism	Gene ID	Gene name	Accession no.
Human	ATP8A2	ATP8A2	Q9NTI2
P. falciparum	PF3D7_1219600	ATP2	Q8I5L4
S. mansoni	Smp_091650	Smp_091650	A0A3Q0KJ05
S. mansoni	Smp_104500	Smp_104500	A0A5K4EK18
S. mansoni	Smp_163820	Smp_163820	A0A5K4EW97
S. mansoni	Smp_181230	Smp_181230	A0A3Q0KTZ9
S. mansoni	Smp_332390	Smp_332390	A0A5K4F868
S. mansoni	Smp_333250	Smp_333250	A0A5K4FA05

B

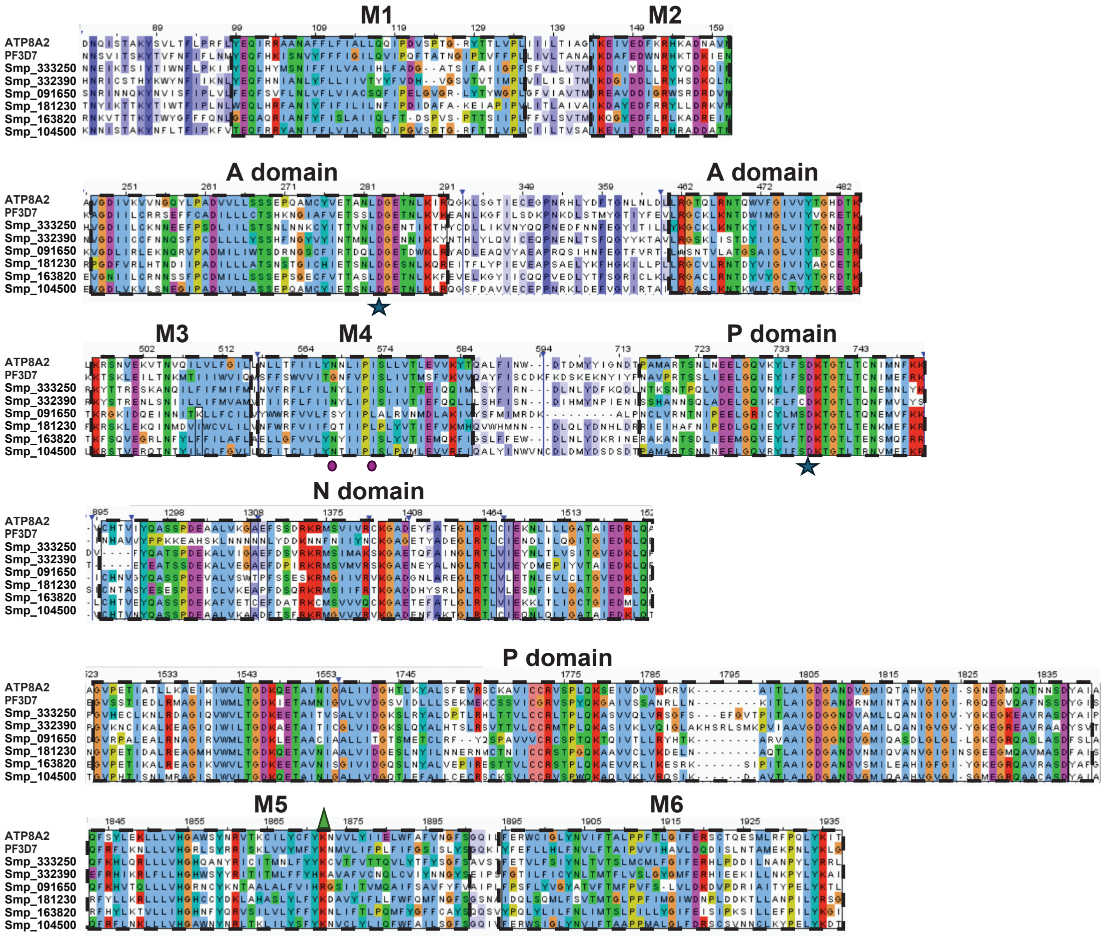

Supplementary Figure 2: Sequence alignment of phospholipid flippases. **A)** Lipid flippases used in the alignment. **B)** Sequence alignment of human ATP8A2, *P. falciparum* PfATP2, and six putative lipid-translocating ATPases from *S. mansoni*. The actuator (A), nucleotide binding (N), and phosphorylation (P) domains are shown, as well as the first six transmembrane segments (M1-6). Key conserved residues D (in A domain) and E (P domain) involved in the phosphorylation (DKTGT) and dephosphorylation (DGET) cycle are highlighted by a star. The purple circles highlight the conserved N and I residues located in M4 domain that are important for recognition and release of lipid, respectively. The green triangle indicates the K residues in the M5 domain required for the sensitivity to the lipid subtype [14, 28].

bioRxiv preprint doi: https://doi.org/10.1101/2024.04.23.590861; this version posted April 24, 2024. The copyright holder for this preprint (which was not certified by peer review) is the author/funder, who has granted bioRxiv a license to display the preprint in perpetuity. It is made available under aCC-BY 4.0 International license.

List of Supplementary Tables

Supplementary Table 1: Compound information.

Supplementary Table 2: Activity against *Plasmodium* asexual blood stages, *Schistosoma* schistosomula and HepG2 cells.

Supplementary Table 3: Single-nucleotide variants for compound **22**-selected clones.

Supplementary Table 4: Copy number variants from compound **22**-selected clones.
